# Supplementary material for: Mechanical vibration does not systematically reduce the tremor in essential tremor patients
Source: Sci Rep. 2019 Nov 11;9:16476. doi: 10.1038/s41598-019-52988-8 (PMC6848159; doi:10.1038/s41598-019-52988-8)
Supplement: Supplementary file 1 — Supplementary information [file 41598_2019_52988_MOESM1_ESM.pdf]

# Mechanical vibration does not systematically reduce the tremor in essential tremor patients. Supplementary material

*Julio Salvador Lora-Millán[1]; *Roberto López-Blanco[2,3,4]; Juan Álvaro Gallego[1]; Antonio Méndez-Guerrero[5]; Jesús González de la Aleja[5]; Eduardo Rocon[1]

1. Centre for Automation and Robotics (CAR), CSIC-UPM, Madrid, Spain.
2. Healthcare Research Institute, Hospital Universitario 12 de Octubre. Madrid Spain.
3. Neurology Section . Hospital Virgen de la Poveda, Villa del Prado, Madrid (Spain).
4. Medicine Department, Faculty of Medicine, Universidad Complutense Madrid (UCM), Spain.
5. Neurology Department. Hospital Universitario 12 de Octubre. Madrid (Spain).

Corresponding author: Eduardo Rocon

Email: e.rocon@csic.es

Phone: +34 91 871 19 00

Fax: + 34 91 871 70 50

*These authors contributed equally to this work.

| ***Patient*** | ***Gender*** | ***Age (years)*** | ***Duration of tremor (years)*** | ***FTM-TRS Postural tremor in hand (0-4)*** | ***Treatment* (mg/day)*** |
|---|---|---|---|---|---|
| *P1* | Male | 87.87 | 37.35 | 3 | Clonazepam 0.5 |
| *P2* | Male | 78.18 | 26.34 | 3 | Primidone 500 |
| *P3* | Male | 76.01 | 15.33 | 2 | - |
| *P4* | Male | 81.39 | 10.35 | 2 | Primidone 500 |
| *P5* | Male | 68.00 | 8.35 | 2 | Propranolol 60<br>Primidone 250 |
| *P6* | Male | 73.42 | 9.47 | 3 | Primidone 250 |
| *P7* | Male | 77.91 | 10.47 | 3 | Zonisamide 300<br>Propranolol 80 |
| *P8* | Male | 77.76 | 4.59 | 2 | Primidone 375 |
| *P9* | Female | 82.51 | 6.59 | 2 | Primidone 625 |
| *P10* | Female | 87.14 | 20.62 | 4 | Propranolol 30<br>Primidone 250 |
| *P11* | Female | 66.13 | 10.62 | 1 | - |
| *P12* | Female | 78.91 | 11.70 | 2 | Zonisamide 100<br>Gabapentine 900 |
| *P13* | Male | 81.04 | 39.72 | 3 | Zonisamide 150 |
| *P14* | Female | 61.67 | 6.75 | 2 | - |
| *P15* | Male | 79.79 | 1.75 | 1 | - |
| *P16* | Male | 75.90 | 0.93 | 1 | - |
| *P17* | Male | 70.49 | 4.94 | 1 | - |
| *P18* | Female | 59.56 | 18.95 | 1 | - |

*Medication was kept stable at least since two weeks before the test performance.

**Supplementary Table 1.** Clinical description of patients.

| ***Patient*** | ***50Hz PreStim vs Stim*** | ***250Hz PreStim vs Stim*** | ***Increasing Freq PreStim vs Stim*** | ***Random Freq PreStim vs Stim*** |
|---|---|---|---|---|
| *P1* | 0.2325 | ~0 | ~0 | 0.8902 |
| *P2* | ~0 | ~0 | ~0 | ~0 |
| *P3* | 0.0127 | ~0 | ~0 | ~0 |
| *P4* | ~0 | ~0 | ~0 | ~0 |
| *P5* | ~0 | 0.7169 | ~0 | 0.1516 |
| *P6* | 0.0001 | 0.2875 | 0.5250 | 0.0013 |
| *P7* | ~0 | ~0 | ~0 | ~0 |
| *P8* | ~0 | ~0 | ~0 | ~0 |
| *P9* | ~0 | ~0 | 0.0014 | 0.4066 |
| *P10* | 0.0003 | ~0 | ~0 | ~0 |
| *P11* | 0.1199 | ~0 | 0.0096 | 0.0038 |
| *P12* | ~0 | ~0 | ~0 | ~0 |
| *P13* | ~0 | 0.0890 | ~0 | ~0 |
| *P14* | 0.0444 | ~0 | ~0 | 0.0105 |
| *P15* | ~0 | 0.0561 | 0.0070 | 0.8663 |
| *P16* | 0.0195 | 0.7237 | ~0 | 0.1774 |
| *P17* | 0.0207 | ~0 | 0.9263 | ~0 |
| *P18* | 0.9070 | ~0 | 0.2545 | ~0 |

**Supplementary Table 2.** Changes in tremor amplitude during each stimulation strategy. Each element is the p-value of a Wilcoxon Rank Sum test between the stimulation epoch ($n$=120) and its corresponding pre-Stim epoch ($n$=60).

| ***Patient*** | ***Combined baseline data vs. 50Hz stim*** | ***Combined baseline data vs. 250Hz stim*** | ***Combined baseline data vs. Increasing Freq stim*** | ***Combined baseline data vs. Random Freq stim*** |
|---|---|---|---|---|
| *P1* | 0.1796 | 0.5669 | 0.0107 | ~0 |
| *P2* | ~0 | ~0 | ~0 | ~0 |
| *P3* | ~0 | ~0 | ~0 | ~0 |
| *P4* | ~0 | ~0 | ~0 | ~0 |
| *P5* | ~0 | ~0 | 0.0509 | ~0 |
| *P6* | 0.0032 | 0.0001 | ~0 | ~0 |
| *P7* | ~0 | ~0 | ~0 | ~0 |
| *P8* | ~0 | ~0 | ~0 | ~0 |
| *P9* | ~0 | ~0 | ~0 | ~0 |
| *P10* | ~0 | ~0 | ~0 | ~0 |
| *P11* | 0.0256 | 0.0091 | ~0 | 0.3097 |
| *P12* | ~0 | ~0 | ~0 | 0.6566 |
| *P13* | ~0 | 0.661 | ~0 | ~0 |
| *P14* | ~0 | 0.7908 | ~0 | ~0 |
| *P15* | ~0 | ~0 | 0.0097 | 0.1117 |
| *P16* | 0.6673 | 0.4617 | 0.0137 | ~0 |
| *P17* | ~0 | 0.0020 | 0.0094 | ~0 |
| *P18* | ~0 | ~0 | 0.0492 | ~0 |

**Supplementary Table 3.** Changes in tremor amplitude during each stimulation strategy. Each element is the p-value of a Wilcoxon Rank Sum test between the stimulation epoch ($n$=120) and the combined baseline ($n$=480).

| ***Patient*** | ***IncreasingFreq*** | | ***RandomFreq*** | |
|---|---|---|---|---|
| | ***p-Value*** | ***$R^2$*** | ***p-Value*** | ***$R^2$*** |
| *P1* | ~0 | 0.8207 | 0.8410 | 0.0062 |
| *P2* | ~0 | 0.8546 | 0.1980 | 0.2241 |
| *P3* | 0.1860 | 0.2350 | 0.3005 | 0.1515 |
| *P4* | 0.0014 | 0.7860 | 0.3327 | 0.1340 |
| *P5* | ~0 | 0.8305 | 0.0604 | 0.4166 |
| *P6* | 0.3786 | 0.1121 | 0.9868 | ~0 |
| *P7* | 0.0012 | 0.7963 | 0.0933 | 0.3500 |
| *P8* | 0.0829 | 0.3687 | 0.6746 | 0.0267 |
| *P9* | 0.3645 | 0.1184 | 0.0181 | 0.5739 |
| *P10* | 0.0204 | 0.5598 | 0.4168 | 0.0961 |
| *P11* | 0.5109 | 0.0641 | 0.5311 | 0.0584 |
| *P12* | 0.2852 | 0.1605 | 0.6971 | 0.0230 |
| *P13* | ~0 | 0.8875 | 0.8240 | 0.0076 |
| *P14* | 0.0048 | 0.7029 | 0.6710 | 0.0273 |
| *P15* | 0.3319 | 0.1343 | 0.6671 | 0.0280 |
| *P16* | 0.2756 | 0.1665 | 0.6970 | 0.0230 |
| *P17* | 0.0068 | 0.6718 | 0.0942 | 0.3845 |
| *P18* | 0.4928 | 0.0696 | 0.6313 | 0.0347 |

**Supplementary table 4**. Fit accuracy for linear models that related vibration frequency and tremor amplitude during the IncreasingFreq and RandomFreq trials (in both cases, $n$=9).

| ***Comparison*** | ***Paired-samples t-test p-value*** |
|---|---|
| *NoStim vs 50Hz* | *0.2195* |
| *NoStim vs 250Hz* | *0.5402* |
| *NoStim vs IncreasingFreq* | *0.0115* |
| *NoStim vs RandomFreq* | *0.1613* |

**Supplementary table 4**. Paired-samples t-test results for comparison of Stim/PreStim ratios under the different stimulation strategies and the basal condition.

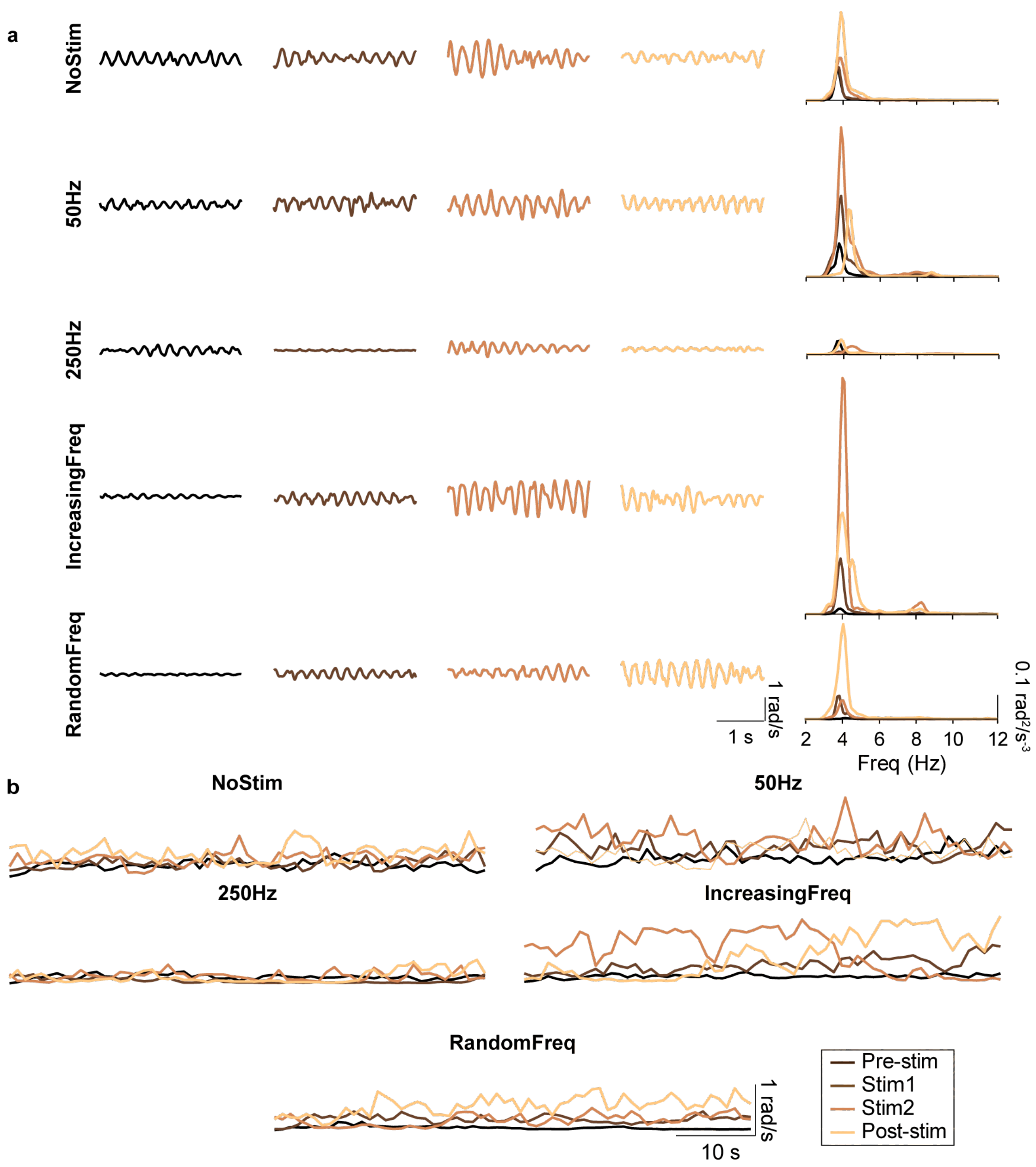


**Supplementary Figure 1.** Example recordings during all stimulation strategies for another representative patient (P12). **a.** Wrist tremor (3s of data) during each of the four epochs (Pre-stim, Stim1, Stim2, Post-stim; each shown in a different column) for all the stimulation strategies (each shown in a different row); the fifth column represents the corresponding power spectral densities (data band-pass filtered between 3-12Hz). **b**. Tremor amplitude during each of the five strategies for the same patient. Each trace represents the time-varying RMS of the tremor amplitude computed in 1 s non-overlapping windows. Same color code as in a.

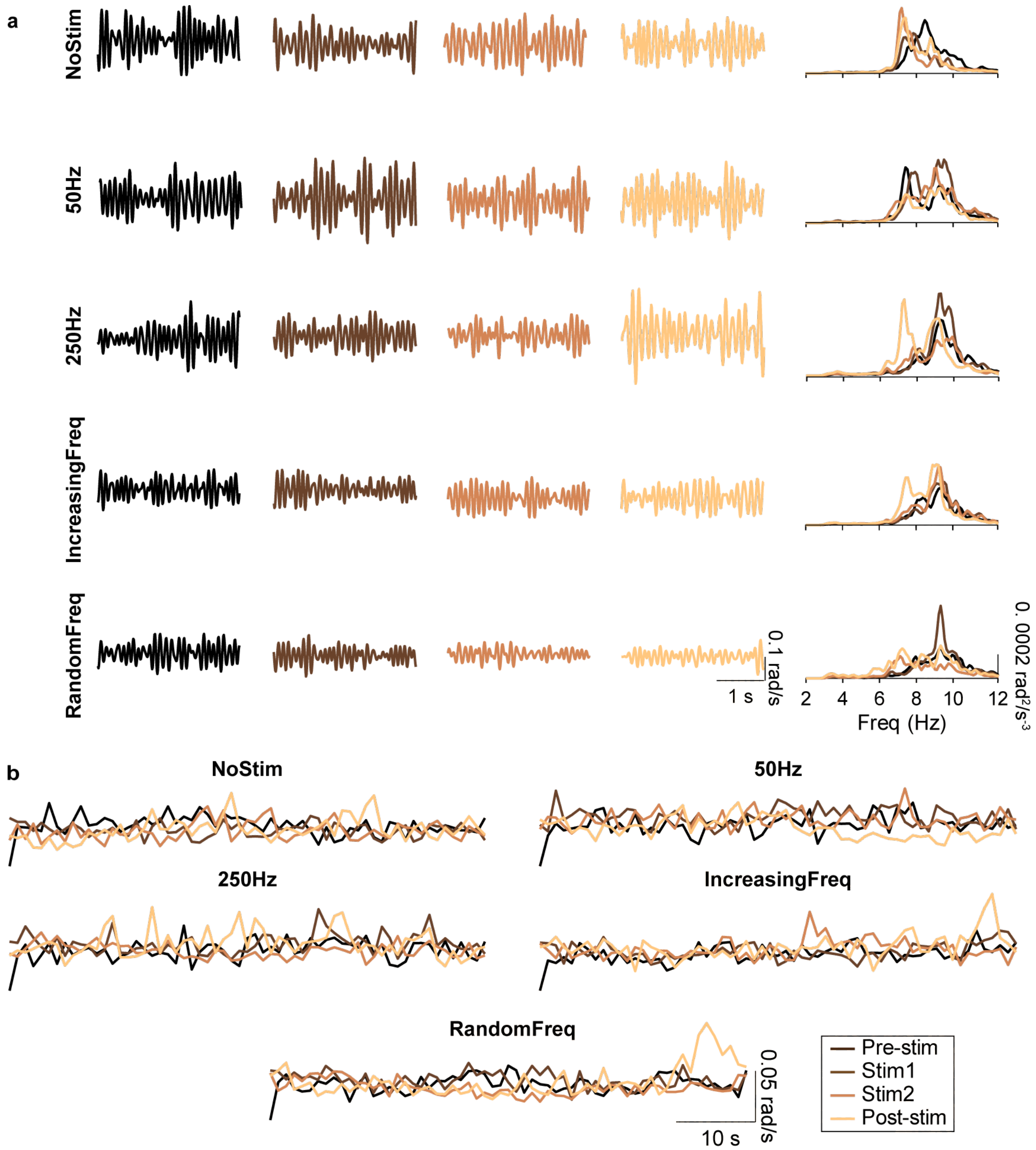


**Supplementary Figure 2.** Example recordings during all stimulation strategies for another representative patient (P15). **a.** Wrist tremor (3s of data) during each of the four epochs (Pre-stim, Stim1, Stim2, Post-stim; each shown in a different column) for all the stimulation strategies (each shown in a different row); the fifth column represents the corresponding power spectral densities (data band-pass filtered between 3-12Hz). **b**. Tremor amplitude during each of the five strategies for the same patient. Each trace represents the time-varying RMS of the tremor amplitude computed in 1 s non-overlapping windows. Same color code as in a.

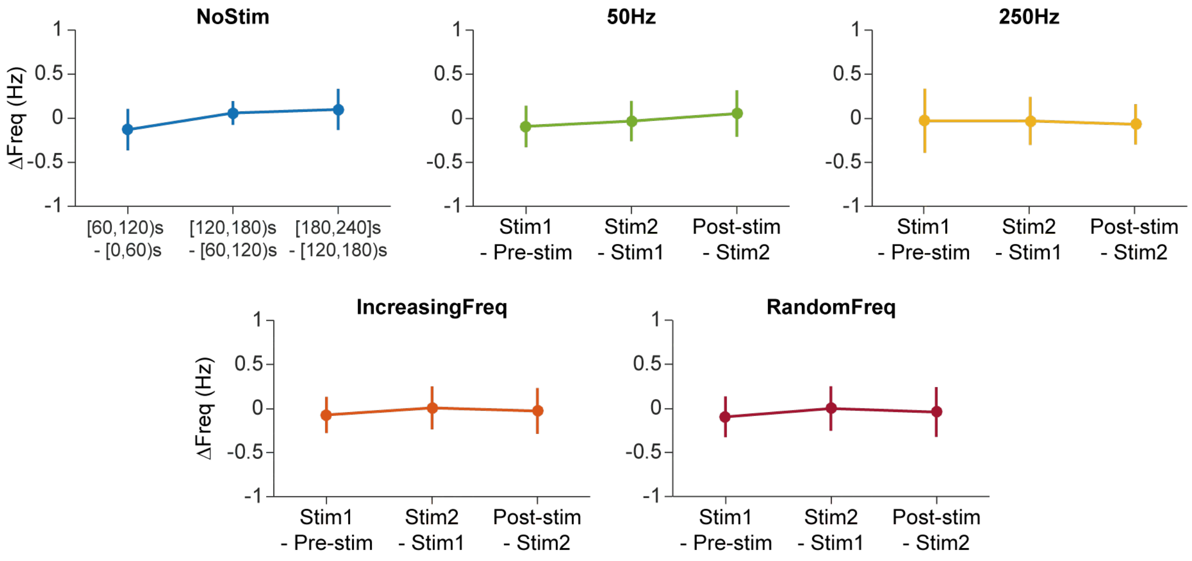


**Supplementary Figure 3.** Tremor frequency remained constant during vibratory stimulation. Each panel shows the mean ± SD change in tremor frequency across consecutive 1-min epochs for each stimulation strategy, pooled over all patients.

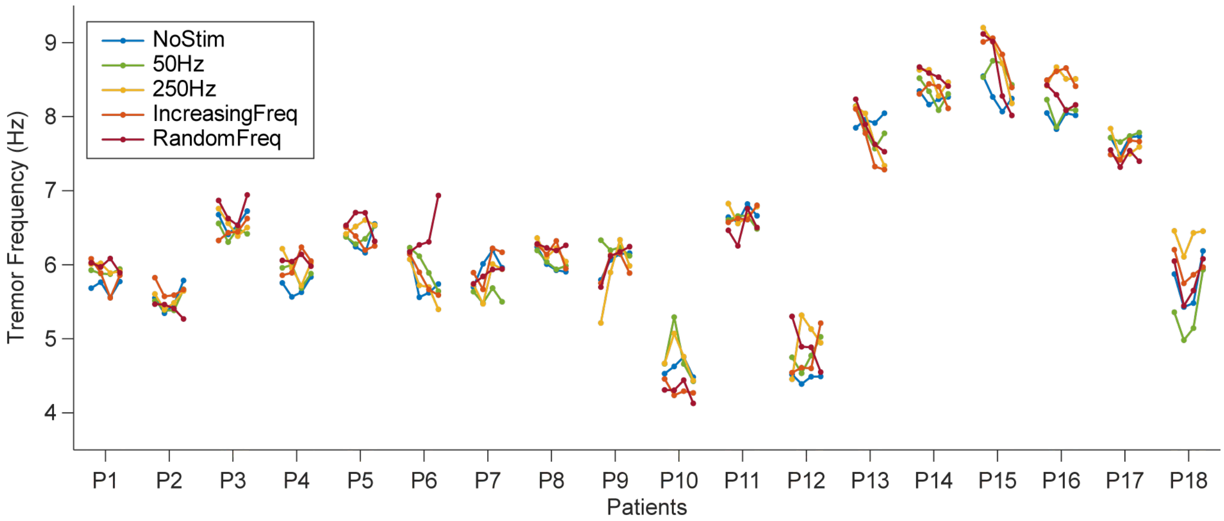


**Supplementary Figure 4.** Summary of the evolution of the frequency tremor across epochs for each patient among stimulation strategies. Each color represents the same stimulation strategy across patients.

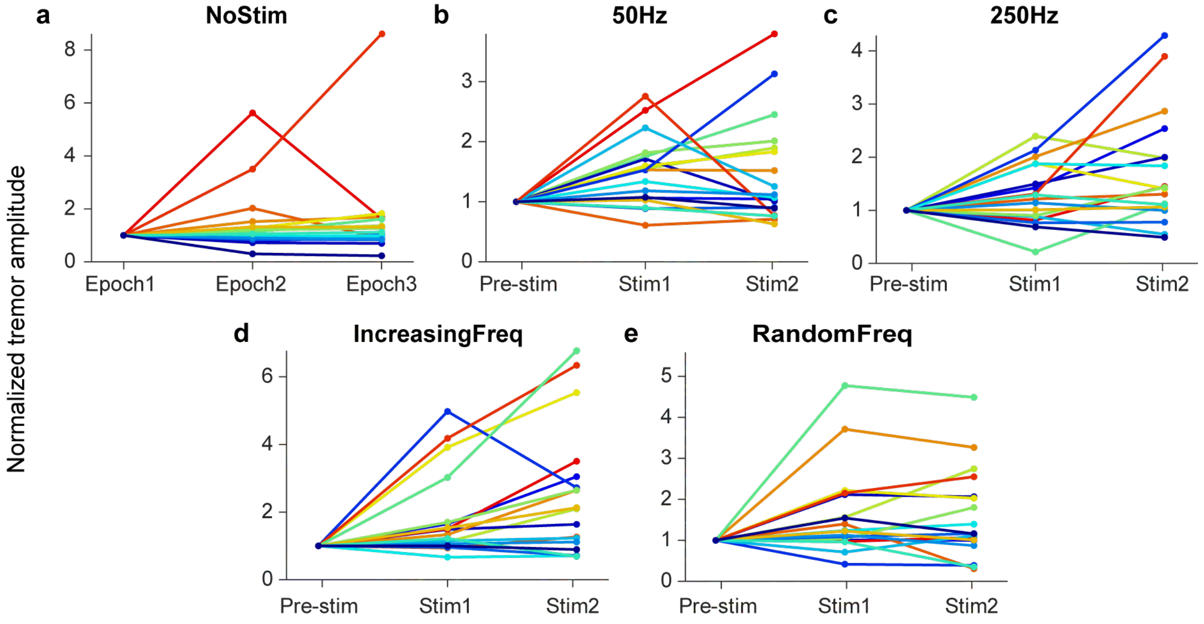


**Supplementary Figure 5**. Normalized Changes in tremor amplitude for each patient, in response to each stimulation strategy. a–e. Median tremor amplitude (RMS) for each strategy (indicated on top) during each 1-min epoch. Each color represents one patient (same color code as in Figure 4).

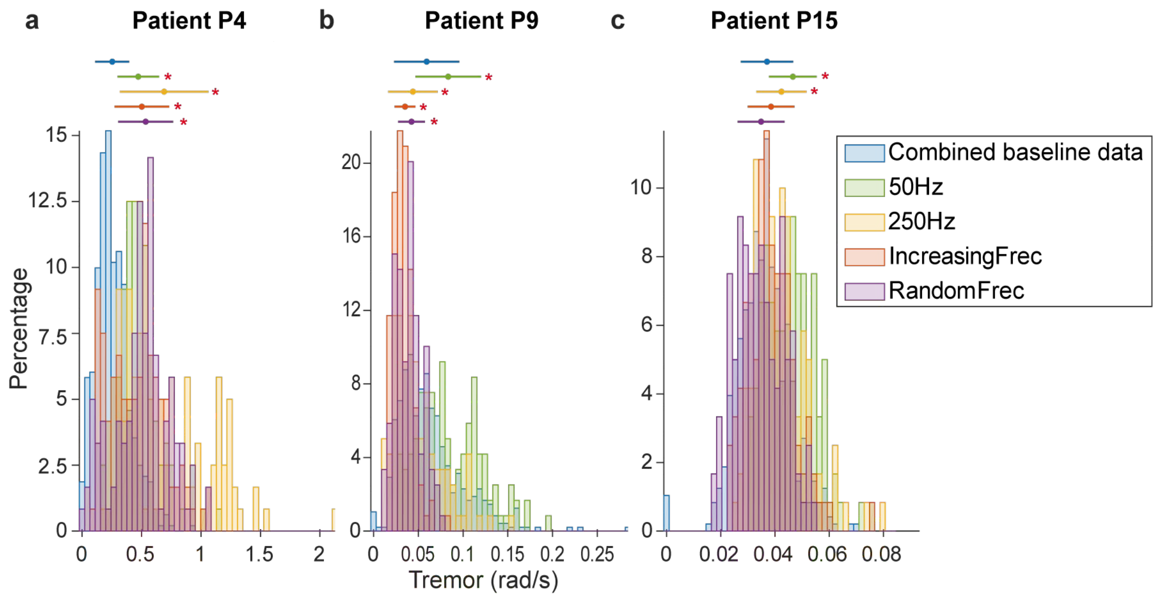


**Supplementary Figure 6.** Effect of the different vibration strategies on a patient's tremor. **a**. Histogram of the combined baseline data tremor (blue) and the tremor during each stimulation strategies (other colors; see legend) for patient P4.. Histogram: tremor amplitude during each 1-s bin of the corresponding stimulation condition. Marker (*) means significant difference respect to the Combined Baseline Data (*P*<0.01). Errorbars: mean +/- SD. **b**, **c**. The same data for patients P9 and P15, respectively.

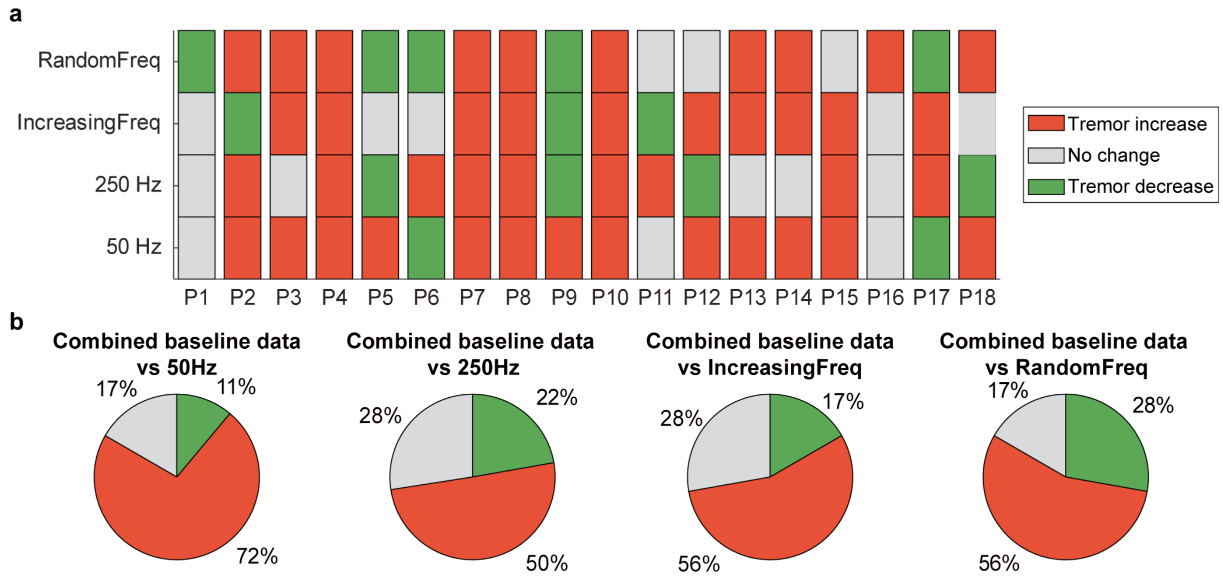


**Supplementary Figure 7.** Summary of the changes in tremor amplitude during the different stimulation strategies, when compared to the combined baseline rather than to the Pre-Stim epoch. **a**. Change in tremor amplitude during each Stim epoch with respect to the combined baseline for each patient, during each trial type. **b.** Percentage of patients for which the tremor decreased, increased or remained unaltered.

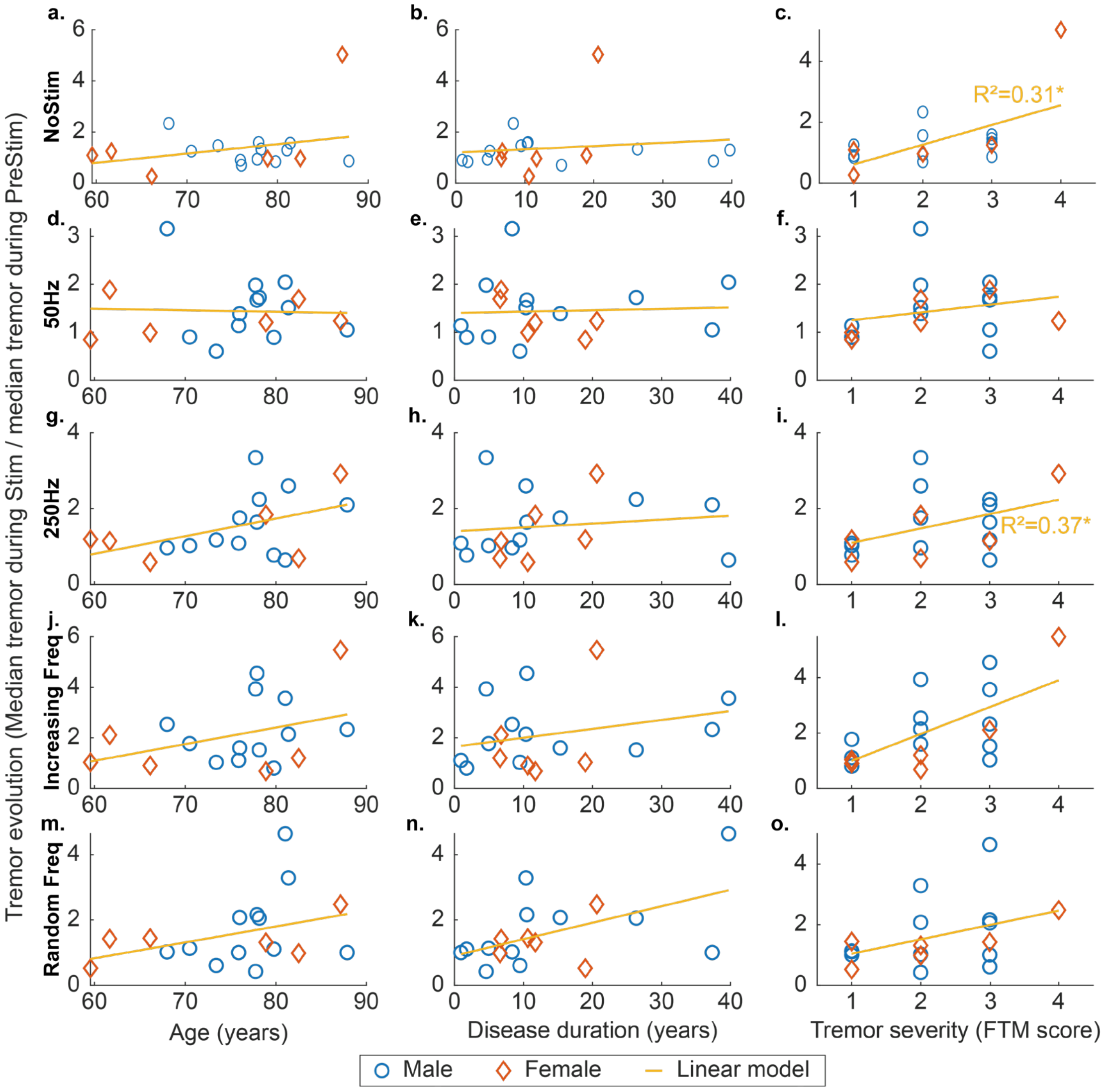


**Supplementary Figure 8.** Relationship between relevant clinical parameters and the observed changes in tremor amplitude. The change in tremor amplitude is computed as the ratio between the median tremor amplitude during the Stim and PreStim epochs (a ratio greater than 1 means that tremor was increased during Stim epoch, while a ratio lower than 1 means that stimulation decreased tremor). Each row shows how the change in tremor amplitude during each of the five experimental conditions relates to a different clinical feature. Yellow line represent the lineal model computed to relate the clinical feature with the tremor behavior; * denotes that the models were statistically significant (P<0.01). Female and male patients are showed using different symbols (legend).
